# Nationwide epidemiology of carbapenem resistant *Klebsiella pneumoniae* isolates from Greek hospitals, with regards to plazomicin and aminoglycoside resistance

**DOI:** 10.1186/s12879-019-3801-1

**Published:** 2019-02-15

**Authors:** Irene Galani, Konstantina Nafplioti, Panagiota Adamou, Ilias Karaiskos, Helen Giamarellou, Maria Souli, Study Collaborators, Sofia Maraki, Viktoria Eirini Mauromanolaki, Vassiliki Papaioannou, Sofia Tsiplakou, Polyzo Kazila, Ioanna Diamanti, Nikoletta Charalampaki, Eleftheria Trikka-Graphakos, Marina Toutouza, Helen Vagiakou, Konstantinos Pappas, Anna Kyratsa, Angeliki Paschali, Konstantina Kontopoulou, Olga Legga, Efthymia Petinaki, Helen Papadogeorgaki, Efrosini Chinou

**Affiliations:** 1 Infectious Diseases Laboratory, 4th Department of Internal Medicine, National and Kapodistrian University of Athens, Faculty of Medicine, Athens, Greece; 2 6th Department of Internal Medicine, Hygeia Hospital, Athens, Greece; 3 University General Hospital “ATTIKON”, Rimini 1, 124 62 Chaidari, Greece

**Keywords:** Plazomicin, *K. pneumoniae*, Carbapenemase, Greece

## Abstract

**Background:**

To evaluate the in vitro activities of plazomicin and comparator aminoglycosides and elucidate the underlying aminoglycoside resistance mechanisms among carbapenemase-producing *K. pneumoniae* isolates collected during a nationwide surveillance study in Greek hospitals.

**Methods:**

Three hundred single-patient carbapenemase-producing *K. pneumoniae* isolates were studied, including 200 KPC-, 50 NDM-, 21 VIM-, 14 KPC & VIM-, 12 OXA-48-, two NDM & OXA- and one KPC & OXA-producing isolates. Susceptibility testing was performed by broth microdilution, and minimum inhibitory concentrations (MICs) interpreted per EUCAST breakpoints. Carbapenemase-, aminoglycoside modifying enzyme- and 16S rRNA methylase- encoding genes were detected by PCR.

**Results:**

Of 300 isolates tested, 5.7% were pandrug resistant and 29.3% extensively drug resistant. Plazomicin inhibited 87.0% of the isolates at ≤2 mg/L, with MIC_50_/MIC_90_ of 0.5/4 mg/L. Apramycin (a veterinary aminoglycoside) inhibited 86.7% of the isolates at ≤8 mg/L and was the second most active drug after plazomicin, followed by gentamicin (S, 43%; MIC_50_/MIC_90_, 4/> 256) and amikacin (S, 18.0%; MIC_50_/MIC_90_, 32/128). Twenty-three (7.7%) isolates (16 KPC-, 6 VIM- and one KPC & OXA-48-producers) exhibited MICs ≥64 mg/L for plazomicin, and harbored *rmtB* (*n* = 22) or *armA* (*n* = 1). AAC(6′)-Іb was the most common aminoglycoside modifying enzyme (84.7%), followed by AAC(3΄)-IIa (25.3%), while those two enzymes were co-produced by 21.4% of the isolates.

**Conclusions:**

Plazomicin retains activity against most carbapenemase-producing *K. pneumoniae* isolated from Greek hospitals, with MICs consistently lower than those of the other aminoglycosides, even in the presence of aminoglycoside modifying enzymes. Dissemination of 16S- rRNA methylases in 8% of the isolates is an unwelcome event that needs strict infection control measures and rigorous stewardship interventions.

**Electronic supplementary material:**

The online version of this article (10.1186/s12879-019-3801-1) contains supplementary material, which is available to authorized users.

## Background

Hospital infections caused by carbapenem-resistant *Klebsiella pneumoniae* constitute a worldwide problem associated with high morbidity, mortality, and prolongation of hospitalization and associated costs [1]. The spread of carbapenemases in *K. pneumoniae* has created therapeutic dilemmas for clinicians as those isolates often demonstrate resistance to many other classes of antibiotics, thus limiting our therapeutic options. Furthermore, few new antibiotics are in line to replace carbapenems [2].

In Greece, carbapenem-resistance *in K. pneumoniae* emerged in 2002 due to carbapenemase production (initially VIM and later KPC, NDM and OXA-48-like) and has become endemic [3]. The current epidemiology of carbapenemase-producing *K. pneumoniae* in Europe has been reported by Grundmann et al., as part of the European Survey on carbapenemase - producing Enterobacteriaceae (EuSCAPE) conducted from November 2013 till April 2014 in 35 European countries [4]. According to this survey, an average of 1.3 patients per 10,000 hospital admissions in Europe had a carbapenemase-producing *K. pneumoniae* or *E. coli* infection, while this incidence specifically in Greece was 5.78, the second highest behind Italy (5.96) [4]. In this survey, among 86 carbapenem-non-susceptible *K. pneumoniae* isolates from Greece, a large proportion -were KPC-positive (65%), followed by NDM (14%), VIM (11%) and OXA-48-positive (2%) [4]. In a recent multi-center study published by our group, among 394 carbapenem-resistant *K. pneumoniae* isolates from 15 Greek hospitals 66.5% were KPC-, 13.7% were NDM-, 8.6% were VIM-, 5.6% were KPC and VIM- and 3.6% were OXA-48-producers [5].

Aminoglycosides are broad-spectrum antibiotics which have been used for the treatment of life-threatening infections. Many mechanisms of acquired resistance to aminoglycosides have emerged, with the aminoglycoside-modifying enzymes (AMEs) being the most prevalent. These enzymes include N-acetyltransferases, O-nucleotidyltransferases and O-phosphotransferases, which inactivate aminoglycosides by covalently modifying specific amino or hydroxyl moieties on the drugs [6]. Another less common mechanism of resistance is the up-regulation of efflux pumps and reduction in membrane permeability developed by bacteria to affect the transport of hydrophilic aminoglycosides across cell membranes. Additionally, 16S rRNA methyltransferases (RMTs), which occur at a low incidence in clinical isolates, modify bacterial 16S rRNA, the molecular target of aminoglycosides and confer high-level resistance to all widely used aminoglycosides [7].

Plazomicin is a next-generation aminoglycoside that was developed to overcome common aminoglycoside-resistance mechanisms for the treatment of patients with serious infections caused by multidrug-resistant Enterobacteriaceae, including ESBL producing and carbapenem-resistant Enterobacteriaceae [8]. Plazomicin is a semi-synthetic derivative of sisomicin, not affected by any known aminoglycoside-modifying enzymes (AMEs), except N-acetyltransferases (AACs) AAC(2′)-Ia, −Ib and -Ic (found only in *Providencia* spp.) [8]. Like sisomicin, it lacks the 3′- and 4′-OH groups, thus is protected from the O-phosphotransferase (APH) APH (3′) and O adenyltransferase (ANT) ANT (4′) enzymes that generate resistance to amikacin. The hydroxy-aminobutyric acid substitute introduced at the N1 position of sisomicin provides protection from the AAC(3), ANT(2″) and APH(2″) AMEs, while the hydroxyethyl substitute at the 6′ position blocks the multitude of AAC(6′) AMEs, without reducing potency, as occurred in previous efforts to shield this position [8]. Plazomicin (ZEMDRI™) was approved in June 2018 by the U.S. Food and Drug Administration, for adults with complicated urinary tract infections (cUTI), including pyelonephritis, caused by certain Enterobacteriaceae in patients who have limited or no alternative treatment options. ZEMDRI is an intravenous infusion, administered once daily.

In this study, we evaluated the in vitro activities of plazomicin and comparator aminoglycosides (amikacin, gentamicin and tobramycin) and elucidated the underlying aminoglycoside resistance mechanisms among 300 carbapenemase-producing *K. pneumoniae* isolates collected during a nationwide surveillance study in Greek hospitals.

## Methods

### Clinical isolates

A total of 300 single-patient carbapenem-non-susceptible *Κ. pneumoniae* isolates, collected between November 2014 and April 2016, from 14 Greek hospitals in six cities, were included. The isolates were collected prior to this study, during a prospective Greek multicenter study, organized by the Hellenic Society of Chemotherapy, and were archived and anonymised.

The mechanisms of carbapenem resistance in these isolates, described in a previous study [5], were production of KPC (*n* = 200; 66.7%), NDM (*n* = 50; 16.7%), OXA-48 (*n* = 13; 4.3%), VIM (*n* = 21; 7.0%), KPC & VIM (*n* = 13; 4.3%), KPC & OXA (*n* = 1; 0.3%) and NDM & OXA (*n* = 2; 0.7%).

Isolates were obtained from specimens of lower respiratory tract (*n* = 51), pus (*n* = 40), CSF (*n* = 2), blood (*n* = 55), urine (*n* = 124) and other sites (*n* = 28). All strains were stored at − 80 °C and were sub-cultured twice before testing.

### Antimicrobial susceptibility testing

Confirmation of the species and MIC determination of ampicillin/sulbactam, piperacillin/tazobactam, cefoxitin, ceftazidime, ceftriaxone, cefepime, aztreonam, imipenem, meropenen, amikacin, gentamicin, ciprofloxacin, levofloxacin, tigecycline, fosfomycin and trimethoprim/sulfamethoxazole was performed by Vitek®2 (bioMérieux, Marcy-l’Etoile, France). Additionally, the MICs of meropenem, tigecycline, and fosfomycin were determined by Etest® (AB Biodisk, Solna, Sweden) according to manufacturer’s instructions and the MIC of colistin was determined by the broth microdilution method according to the Clinical and Laboratory Standards Institute (CLSI) guidelines [9].

Susceptibility testing of plazomicin (Achaogen Inc., South San Francisco, CA, USA), amikacin (Discovery-Fine Chemicals, Wimborne, UK), gentamicin (Applichem, GmbH, Darmstadt, Germany), tobramycin (Serva Electrophoresis GmbH, Heidelberg, Germany), netilmicin (Sigma-Aldrich, St. Louis, MO), neomycin (Applichem) and apramycin (Sigma-Aldrich), was performed by the broth microdilution method according to the Clinical and Laboratory Standards Institute (CLSI) guidelines [9]. *Escherichia coli* ATCC 25922 and *Pseudomonas aeruginosa* ATCC27853 were used as quality control (QC) strains. Results were considered valid if both QC strains tested in each experiment fell within the CLSI designated QC MIC ranges for amikacin, gentamicin, tobramycin and netilmicin [10], and the NCCLS QC MIC ranges for apramycin [11]. Antimicrobial susceptibility results were interpreted according to the EUCAST recommendations (EUCAST 2018, version 8) [12]. For plazomicin the recently approved by the US Food and Drug Administration (FDA) breakpoints (S; ≤2 mg/L, R; ≥8 mg/L) were applied (https://www.fda.gov/Drugs/DevelopmentApprovalProcess/DevelopmentResources/ucm611779.htm). Susceptibility breakpoints for neomycin are not established by EUCAST or CLSI, while apramycin categorical breakpoints (S; ≤8 mg/L, R; ≥64 mg/L) were based on the National Antibiotic Resistance Monitoring Study (NARMS) report (National Antibiotic Resistance Monitoring System (NARMS) Working Group, 2001).

### Molecular characterization of aminoglycoside resistance mechanisms

All isolates demonstrating non-susceptibility to at least one of the tested aminoglycosides were screened for the presence of AME genes, while isolates demonstrating simultaneously high-level resistance (≥128 mg/L) to amikacin, gentamicin, netilmicin and tobramycin, were further tested for RMT-encoding genes by PCR. Template DNA was extracted from bacteria grew in Luria Bertani broth for 18 h by the use of NucleoSpin Tissue kit (Machery-Nagel GmbH, Duren, Germany). Screening for AME genes *aac(6′)-Ib, aac(3′)-IIa, aac(3′)-Ia, aac(3′)-IV, aph(3′)-VI, ant(2′)-Ia* and *ant(3′)-I*, was performed by simplex ‘in-house’ PCR assays and for RMT coding genes *armA, rmtA, rmtB, rmtC, rmtD/rmtD2, rmtE, rmtF, rmtG, rmtH* and *npmA,* by two multiplex PCR protocols with specific primers and conditions (see Additional file 1: Table S1).

### Typing

Genetic relatedness among carbapenemase producing *K. pneumoniae* isolates was evaluated by pulsed-field gel electrophoresis (PFGE) analysis of chromosomal restriction fragments obtained following cleavage with *Spe*I (New England BioLabs Inc., GmbH Frankfurt am Main, Germany). A dendrogram was generated from the homology matrix with a coefficient of 1.5% using the unweighted pair-group method using arithmetic averages (“UPGMA”) to describe the relationships among PFGE profiles. Isolates were considered to belong to the same PFGE group if their Dice similarity index was ≥80%.

## Results

Of 300 isolates tested, 17 were pandrug-resistant (PDR) (5.7%), 88 were extensively drug-resistant (XDR) (29.3%) and the remaining 195 were multi-drug resistant (MDR) (65.0%), according to the definitions provided by the ECDC [13]. Isolates were highly resistant not only to carbapenems (MIC_90_ > 8 mg/L) and piperacillin-tazobactam (MIC_90_ > 64/4 mg/L) but also to ciprofloxacin (MIC_90_ > 2 mg/L). Aztreonam was active against 29 isolates (9.7%), all producing an MBL carbapenemase (VIM or NDM) and trimethoprim/sulfamethoxazole against 71 isolates (23.7%). Colistin was the most active drug in vitro, with 66.7% of the isolates being susceptible (MIC_50_/MIC_90_, 1/32 mg/L), followed by fosfomycin (S, 62.7%, MIC_50_/MIC_90_, 32/256 mg/L). Finally, tigecycline demonstrated 53.0% susceptibility with an MIC_50_ of 1 and an MIC_90_ of 4 mg/L.

Only twenty-four *Κ. pneumoniae* isolates (8.0%) were aminoglycoside-susceptible, whereas the remaining 276 isolates (92.0%) were resistant to at least one of the indicated aminoglycosides tested, i.e. amikacin, gentamicin, tobramycin or netilmicin, using the clinical and epidemiological breakpoints defined by EUCAST. Gentamicin was the most active in vitro aminoglycoside in clinical use, with 43.0% being susceptible, followed by amikacin (S, 18.0%). The highest resistance rates were observed for tobramycin, with 89 and 83.3% resistant according to EUCAST and CLSI breakpoints, and netilmicin, with 87.3 and 84.3% respectively. Finally, 154 (51.3%) and 80 (26.7%) isolates were non-susceptible and resistant, respectively, to the four clinically available aminoglycosides (amikacin, gentamicin, tobramycin and netilmicin) per EUCAST breakpoints. 79 (26.3%) and 43 (14.3%) were non-susceptible and resistant, respectively, when CLSI breakpoints were applied. The in vitro activity of tested aminoglycosides against the studied collection of 300 clinical isolates is presented in Table 1.

**Table 1:** In vitro activity of 7 aminoglycosides against 300 carbapenem-non susceptible *K. pneumoniae* in regards to carbapenemase production

Antimicrobial agent	MIC (mg/L)			Clinical breakpoint (mg/L)		% Susceptible	
	Range	MIC_50_	MIC_90_	EUCAST	CLSI	EUCAST	CLSI
All (*n* = 300)							
Plazomicin	0.125 - > 256	0.5	4	N/A	N/A	87.00% ^a^	
Amikacin	1- > 256	32	128	≤8	≤16	18.00%	31.33%
Gentamicin	0.25- > 256	4	> 256	≤2	≤4	43.00%	61.33%
Tobramycin	0.25- > 256	32	256	≤2	≤4	8.67%	11.00%
Netilmicin	0.25- > 256	128	> 256	≤2	≤8	9.00%	13.00%
Neomycin	0.25- > 256	16	> 256	≤8^b^	N/A	43.67% ^a^	N/A
Apramycin	1-128	8	16	≤8^c^	N/A	86.67% ^c^	
KPC (*n* = 201)^d^							
Plazomicin	0.125 - > 256	1	4	N/A	N/A	84.58% ^a^	
Amikacin	1- > 256	32	128	≤8	≤16	17.91%	31.34%
Gentamicin	0.25- > 256	4	64	≤2	≤4	48.76%	71.14%
Tobramycin	0.25- > 256	32	256	≤2	≤4	11.94%	14.93%
Netilmicin	0.25- > 256	128	> 256	≤2	≤8	11.94%	16.92%
Neomycin	0.5- > 256	32	> 256	≤8^b^	N/A	31.34% ^b^	N/A
Apramycin	1-128	4	16	≤8^c^	N/A	86.57% ^c^	
NDM (*n* = 52)^e^							
Plazomicin	0.125 - 2	0.5	1	N/A	N/A	100.00% ^a^	
Amikacin	8-256	32	64	≤8	≤16	17.31%	44.23%
Gentamicin	1- > 256	64	> 256	≤2	≤4	28.85%	36.54%
Tobramycin	16- > 256	32	128	≤2	≤4	0.00%	0.00%
Netilmicin	8- > 256	128	> 256	≤2	≤8	0.00%	1.92%
Neomycin	1-32	2	8	≤8^b^	N/A	94.23% ^b^	N/A
Apramycin	2-32	4	8	≤8^c^	N/A	94.23% ^c^	
VIM (*n* = 21)							
Plazomicin	0.125 - > 256	1	> 256	N/A	N/A	71.43% ^a^	
Amikacin	1- > 256	32	> 256	≤8	≤16	19.05%	42.86%
Gentamicin	1- > 256	128	> 256	≤2	≤4	38.10%	42.86%
Tobramycin	4- > 256	32	> 256	≤2	≤4	0.00%	4.76%
Netilmicin	0.25- > 256	64	> 256	≤2	≤8	0.00%	0.00%
Neomycin	0.5- > 256	32	> 256	≤8^b^	N/A	33.33% ^b^	N/A
Apramycin	2-64	4	16	≤8^c^	N/A	80.95% ^c^	
OXA-48 (*n* = 12)							
Plazomicin	0.125 - 4	0.5	2	N/A	N/A	91.67% ^a^	
Amikacin	2-64	16	32	≤8	≤16	33.33%	58.33%
Gentamicin	1-256	128	256	≤2	≤4	16.67%	16.67%
Tobramycin	0.5-64	64	64	≤2	≤4	16.67%	16.67%
Netilmicin	0.5-256	128	256	≤2	≤8	16.67%	16.67%
Neomycin	0.25-256	2	16	≤8^b^	N/A	83.33% ^b^	N/A
Apramycin	4-8	8	8	≤8^c^	N/A	100.00% ^c^	
KPC and VIM (*n* = 14)							
Plazomicin	0.25 - 2	1	2	N/A	N/A	92.86% ^a^	
Amikacin	4- > 256	64	256	≤8	≤16	7.14%	28.57%
Gentamicin	1-64	4	8	≤2	≤4	42.86%	78.57%
Tobramycin	8- > 256	32	128	≤2	≤4	0.00%	0.00%
Netilmicin	4- > 256	128	> 256	≤2	≤8	0.00%	7.14%
Neomycin	0.5- > 256	> 256	> 256	≤8^b^	N/A	14.29% ^b^	N/A
Apramycin	2-32	8	32	≤8^c^	N/A	57.14% ^c^	

N/A, not available (breakpoints have not been established)

^a^ For plazomicin, the breakpoint recently approved by the FDA was used

^b^ For neomycin, EUCAST epidemiological cut-off values (ECOFFs) for *E.coli* were used

^c^ For apramycin, epidemiological breakpoints from the European Food Safety Authority (EFSA) were used

^d^ One isolate co-producing KPC and OXA-48-like is included

^e^ Two isolates co-producing NDM and OXA-48-like are included

Plazomicin MICs ranged from 0.125 to > 256 mg/L, with MIC_50_ and MIC_90_ of 0.5 and 4 mg/L, respectively. Of note, 87.0% of the isolates were inhibited by plazomicin at ≤2 mg/L, which is the breakpoint approved by the FDA, and 91.3% at ≤4 mg/L. Plazomicin was the most active aminoglycoside tested with an MIC_90_ value ≥32 times lower than that of all aminoglycosides in clinical use tested, > 64 times lower than neomycin and 4 times lower than apramycin (Table 1). Among isolates that were non-susceptible or resistant to the four aminoglycosides in clinical use (according to the EUCAST breakpoints), plazomicin exhibited an MIC_50_ of 1 and 2 mg/L, respectively. The activities of the aminoglycosides against all isolates as well as isolates categorized according to the specific carbapenemase produced are summarized in Table 1. Further, the plazomicin MIC distribution and the cumulative percentage inhibited, in relation to the carbapenemase produced and in relation to amikacin and/or gentamicin susceptibility are presented in Tables 2 and 3, respectively.

**Table 2:** MIC and cumulative percent inhibited distributions for plazomicin, in relation to the carbapenemase type produced by the 300 *K. pneumoniae* isolates

Carbapenemase	No of isolates	No of isolates / (cumulative % of isolates) inhibited at plazomicin MIC (mg/L) of ^a^									
		≤0.125	0.25	0.5	1	2	4	8	16	32	≥64^b^
KPC	200	3 (1.5)	46 (24.5)	46 (47.5)	57 (76.0)	18 (85.0)	**11** **(90.5)**	3 (92.0)	0 (92.0)	0 (92.0)	16 (100)
NDM	50	1 (2.0)	23 (48.0)	13 (74.0)	**11** **(96.0)**	2 (100)	0 (−)	0 (−)	0 (−)	0 (−)	0 (−)
VIM	21	3 (14.3)	3 (28.6)	3 (42.9)	5 (66.7)	1 (71.4)	0 (71.4)	0 (71.4)	0 (71.4)	0 (71.4)	**6** **(100)**
OXA-48	12	1 (8.3)	2 (25.0)	5 (66.7)	2 (83.3)	**1** **(91.7)**	1 (100)	0 (−)	0 (−)	0 (−)	0 (−)
KPC + VIM	14	0 (0.0)	2 (14.3)	0 (14.3)	8 (71.4)	**3** **(92.9)**	1 (100)	0 (−)	0 (−)	0 (−)	0 (−)
NDM + OXA-48	2	0 (0.0)	2 (100)	0 (−)	0 (−)	0 (−)	0 (−)	0 (−)	0 (−)	0 (−)	0 (−)
KPC + OXA-48	1	0 (0.0)	0 (0.0)	0 (0.0)	0 (0.0)	0 (0.0)	0 (0.0)	0 (0.0)	0 (0.0)	0 (0.0)	1 (100)
All	300	8 (2.7)	78 (28.7)	67 (51.0)	83 (78.7)	25 (87.0)	**13** **(91.3)**	3 (92.3)	0 (92.3)	0 (92.3)	23 (100)

^a^ The MIC inhibiting 90% of isolates (MIC_90_) is bolded

^b^ All isolates (*n* = 23) with plazomicin MIC ≥64 mg/L harbored a 16S rRNA methylase gene

**Table 3:** MIC and cumulative percent inhibited distributions for plazomicin, in relation to gentamicin and amikacin susceptibility (according EUCAST criteria)

Aminoglycoside susceptibility	No of isolates	No of isolates / (cumulative % of isolates) inhibited at plazomicin MIC (mg/L) of ^a^									
		≤0.125	0.25	0.5	1	2	4	8	16	32	≥64^d^
Amikacin S^b^	54	5 (9.3)	23 (51.9)	11 (72.2)	9 (88.9)	**1** **(90.7)**	5 (100)	0 (−)	0 (−)	0 (−)	0 (−)
Amikacin NS^c^	246	3 (1.2)	55 (23.6)	56 (46.3)	74 (76.4)	24 (86.2)	8 (89.4)	**3** **(90.7)**	0 (90.7)	0 (90.7)	23 (100)
Amikacin NS (excluding RMT positive isolates)	223	3 (1.3)	55 (26.0)	56 (51.1)	74 (84.3)	**24** **(95.1)**	8 (98.7)	3 (100)	0 (−)	0 (−)	0 (−)
Gentamicin S	129	3 (2.3)	52 (42.6)	41 (74.4)	**24** **(93.0)**	5 (96.9)	4 (100)	0 (−)	0 (−)	0 (−)	0 (−)
Gentamicin NS	171	5 (2.9)	26 (18.1)	26 (33.3)	59 (67.8)	20 (79.5)	9 (84.8)	3 (86.5)	0 (86.5)	0 (86.5)	**23** **(100)**
Gentamicin NS (excluding RMT positive isolates)	148	5 (3.4)	26 (20.9)	26 (38.5)	59 (78.4)	**20** **(91.9)**	9 (97.8)	3 (100)	0 (−)	0 (−)	0 (−)
Amikacin NS/Gentamicin NS	154	2 (1.3)	15 (11.0)	25 (27.3)	58 (64.9)	20 (77.9)	8 (83.1)	3 (85.1)	0 (85.1)	0 (85.1)	**23** **(100)**
Amikacin NS/Gentamicin NS (excluding RMT positive isolates)	131	2 (1.5)	15 (13.0)	25 (32.1)	58 (76.3)	**20** **(91.6)**	8 (97.7)	3 (100)	0 (−)	0 (−)	0 (−)
Amikacin NS/Gentamicin S	92	1 (1.1)	40 (44.6)	31 (78.3)	**16** **(95.7)**	4 (100)	0 (−)	0 (−)	0 (−)	0 (−)	0 (−)
Amikacin S/Gentamicin NS	17	3 (17.6)	11 (82.4)	1 (88.2)	**1** **(94.1)**	0 (94.1)	1 (100)	0 (−)	0 (−)	0 (−)	0 (−)
Amikacin S/Gentamicin S	28	1 (3.6)	8 (32.1)	6 (53.6)	8 (82.1)	1 (85.7)	**4** **(100)**	0 (−)	0 (−)	0 (−)	0 (−)
All isolates excluding RMT positives	277	8 (2.9)	78 (31.0)	67 (55.2)	83 (85.2)	**25** **(94.2)**	13 (98.9)	3 (100)	0 (−)	0 (−)	0 (−)

^a^ The MIC inhibiting 90% of isolates (MIC_90_) is bolded

^b^ S; susceptible

^c^ NS; non susceptible

^d^ All isolates (*n* = 23) with plazomicin MIC ≥64 mg/L harbored a 16S rRNA methylase gene

Twenty-three strains (7.7%), isolated in seven of the 14 hospitals, were highly resistant to all indicated aminoglycosides (MICs ≥256 mg/L), had highly elevated plazomicin MICs (≥64 mg/L) and harbored a RMT gene (Tables 4 and 5). Fifteen KPC-, 6 VIM- and one KPC & OXA-48 -producing *K. pneumoniae* isolates harbored *rmtB*, and one KPC-producing *K. pneumoniae* isolate harbored *armA*. It is of note that none of the NDM- producing *K. pneumoniae* isolates produced a RMT although *bla*_NDM_-carrying plasmids frequently are associated with *armA, rmtB, rmtC*, and *rmtF* [7].

**Table 4:** Presence of aminoglycoside-modifying enzyme gene combination in relation to carbapenemase gene content

AME gene(s)	Carbapenemase gene	*bla*_KPC_	*bla*_NDM_	*bla*_VIM_	*bla*_OXA-48_	*bla*_KPC_ + *bla*_VIM_	*bla*_NDM_ + *bla*_OXA-48_	*bla*_KPC_ + *bla*_OXA-48_
	No (%) of isolates	(*n* = 200)	(*n* = 50)	(*n* = 21)	(*n* = 12)	(*n* = 14)	(*n* = 2)	(*n* = 1)
None	21 (7.0)	19 (9.5)			2 (16.7)			
*aac(6΄)-Ib*	42 (14.0)	24 (12.0)	17 (34.0)		1 (8.3)			
*aac(6′)-Ib + ant(3′)-I*	12 (4.0)	9 (4.5)		3 (14.3)				
*aac(6΄)-Ib + aph(3΄)-Ia*	28 (9.3)	27 (13.5)				1 (7.1)		
*aac(6΄)-Ib + aph(3΄)-Ia + ant(3′)-I*	86 (28.7)	71 (35.5)		4 (19.0)		11 (78.6)		
*aac(3΄)-IIa*	3 (1.0)		2 (4.0)		1 (8.3)			
*aac(3′)-IIa, ant(3′)-I*	2 (0.7)			2 (9.5)				
*aac(3′)-IIa, aph(3′)-Ia, ant(3′)-I*	1 (0.3)						1 (*)	
*aac(6΄)-Ib + aac(3΄)-IIa*	32 (10.7)	1 (0.5)	28 (56.0)		2 (16.7)		1 (*)	
*aac(6΄)-Ib + aac(3΄)-IIa, ant(3′)-I*	5 (1.7)	1 (0.5)			4 (33.3)			
*aac(6΄)-Ib + aac(3΄)-IIa + aph(3΄)-Ia*	8 (2.7)	5 (2.5)	1 (2.0)		2 (16.7)			
*aac(6΄)-Ib + aac(3΄)-IIa + aph(3΄)-Ia, ant(3′)-I*	19 (6.3)	17 (8.5)		2 (9.5)				
*aph(3΄)-Ia*	1 (0.3)	1 (0.5)						
*aph(3′)-Ia, ant(3′)-I*	2 (0.7)	2 (1.0)						
*ant(3′)-I*	4 (1.3)	2 (1.0)		1 (4.8)		1 (7.1)		
*rmtB + any AME*	22 (7.3)	15 (7.5)		6 (28.6)				1 (*)
*armA, aac(6′)-Ib, aph(3′)-Ia, ant(3′)-I*	1 (0.3)	1 (0.5)						
*aac(6′)-Ib + ant(2′)-Ia + ant(3′)-I*	1 (0.3)					1 (7.1)		
*aac(6′)-Ib + ant(2′)-Ia + aph(3′)-Ia*	1 (0.3)	1 (0.5)						
*aac(6′)-Ib + aph(3′)-VI*	4 (1.3)	3 (1.5)	1 (2.0)					
*aac(6′)-Ib + aph(3′)-Ia + aph(3′)-VI*	1 (0.3)	1 (0.5)						
*aac(6′)-Ib + aph(3′)-VI + ant(3′)-I*	1 (0.3)			1 (4.8)				
*aac(6′)-Ib + aac(3′)-IIa + aph(3′)-VI*	1 (0.3)		1 (2.0)					
*aac(6′)-Ib + aac(3′)-IIa + ant(3′)-I+ aph(3′)-Ia + aph(3′)-VI*	2 (0.7)			2 (9.5)				
*aac(6΄)-Ib ± any other AME/RMT*	254 (84.7)	168 (84.0)	48 (96.0)	15 (71.4)	9 (75.0)	13 (92.9)	1 (*)	
*aac(3΄)-IIa ± any other AME/RMT*	76 (25.3)	26 (13.0)	32 (64.0)	7 (33.3.0)	9 (75.0)		2 (*)	
*aph(3΄)-Ia ± any other AME/RMT*	167 (55.7)	137 (68.5)	1 (2.0)	14 (66.7)	2 (16.7)	12 (85.7)	1 (*)	
*ant(3′)-I ± any other AME/RMT*	156 (52.0)	126 (63.0)		21 (100.0)	4 (33.3)	13 (92.9)	1 (*)	1 (*)
*aph(3′)-VI ± any other AME/RMT*	9 (3.0)	4 (2.0)	2 (4.0)	3 (14.3)				
*ant(2′)-Ia ± any other AME/RMT*	3 (1.0)	1 (0.5)		1 (4.8)		1 (7.1)		

* % of the isolates was not determined because of the low numbers of isolates

**Table 5:** Aminoglycoside MICs in relation to the presence of aminoglycoside-modifying enzyme gene combination (excluding the presence of *ant(3΄)-I* and *aph(3′*)-I

AME gene(s)	No (%) of isolates	Expected resistance phenotype^a^	Amikacin			Gentamicin			Tobramycin			Plazomicin		
			Range	MIC_50/90_	% NS	Range	MIC_50/90_	% NS	Range	MIC_50/90_	% NS	Range	MIC_50/90_	% NS^b^
None	28 (9.3)	Susceptible	1-8	2/8	0.0	0.25-4	1/2	7.1	0.25-8	1/4	10.7	≤0.125-4	0.5/4	14.3
*aac(6΄)-Ib*	168 (66)	A,T,N	2-256	32/128	91.7	0.25- > 256	2/16	47.0	2- > 256	32/64	99.4	≤0.125-8	0.5/2	4.2
*aac(3΄)-IIa*	6 (2.0)	G,T,N	2-8		0.0	64-256		100.0	8-64		100.0	≤0.125-1		0.0
*aac(6΄)-Ib + aac(3΄)-IIa*	64 (21.3)	A,G,T,N	4-256	32/64	90.6	1- > 256	64/256	81.3	4- > 256	64/128	100.0	≤0.125-8	0.5/1	4.7
*aac(6′)-Ib + aph(3′)-VI*	6 (2.0)	A,T,N	32-128		100.0	2-64		66.7%	4-256		100.0	0.25-4		33.3
*aac(6′)-Ib + aac(3′)-IIa + aph(3′)-VI*	3 (1.0)	A,G,T,N	32-256		100.0	128- > 256		100.0	32-256		100.0	0.25-1		0.0
*aac(6′)-Ib + ant(2′)-Ia*	2 (0.7)	A,G,T,N	16-128		100.0	8		100.0	32		100.0	0.25-2		0.0
*rmtB or armA ± any AME*	23 (7.7)	A,G,T,N,P	> 256	> 256	100	128- > 256	> 256	100.0	64- > 256	> 256	100.0	64- > 256	> 256	100.0

^a^ A, resistance to amikacin; G, resistance to gentamicin; T, resistance to tobramycin; N, resistance to netilmicin; P, resistance to plazomicin

^b^ According the recently approved FDA breakpoint

For strains carrying no RMT gene (*n* = 277), the MIC_90_ of plazomicin was 2 mg/L, with 94.2% of isolates being susceptible and the highest MIC observed at 8 mg/L.

Among those 277 isolates, 148 (53.4%) and 225 (80.5%) were non-susceptible to gentamicin and amikacin, respectively. One hundred thirty-one (47.3%) isolates were non-susceptible to both gentamicin and amikacin and against 120 (91.6%) of these, the MIC of plazomicin was ≤2 mg/L (Table 3). Isolates with plazomicin MIC 8 mg/L (*n* = 3) were non-susceptible to all other aminoglycosides tested including apramycin.

The most common AME gene was *aac(6′)-Ib* (254 strains; 84.7%), followed by *aph(3′)-Ia* (167 strains; 55.7%), *ant(3′)-Ia* (156 strains; 52.0%), and *aac(3′)-IIa* (76 strains; 25.3%). Nine isolates harboured *aph(3′)-*VIa (3.0%) and three isolates *ant(2′)-Ia* (1.0%), while all isolates were negative for *aac(3)-Ia* and *aac(3)-IVa* (Table 4).

**Fig. 1:**
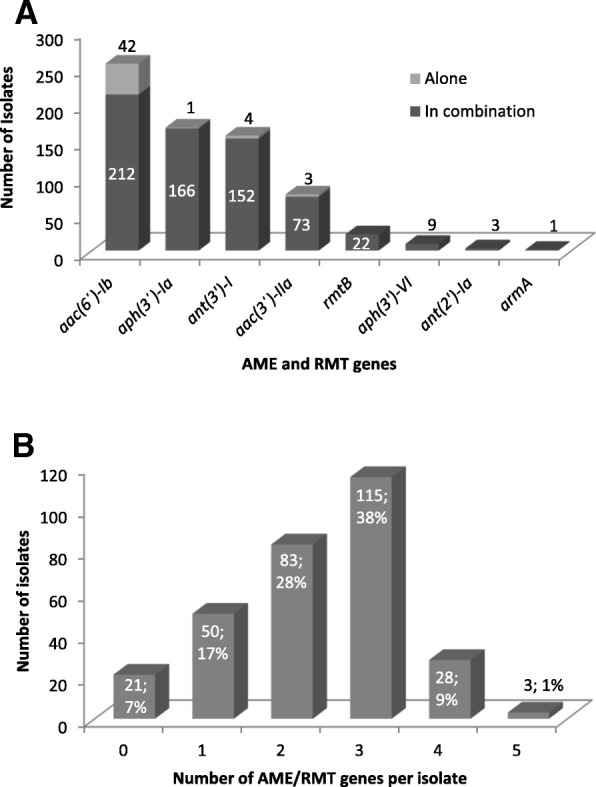
Aminoglycoside-modifying enzymes (AMEs) and 16S rRNA methylases (RMTs) in carbapenemase-producing *K. pneumoniae* isolates. **a** Distribution of AMEs and RMTs. AMEs and RMTs that were present alone and those that were present in combination with other AMEs/RMTs are indicated by gray and black bars, respectively. **b** Number of AMEs/RMTs possessed by the isolates

The majority of isolates harbored at least two (83 isolates; 27.7%) or more AME genes (146 isolates; 48.7%), while less commonly observed were isolates with one AME gene (50 isolates; 16.7% or no AME gene (21 isolates; 7%) (Fig. 1b). The combination of *aac(6′)-Ib, ant(3′)-I* and *aph(3′)-Ia* was the most common (86 isolates; 28.7%), followed by *aac(6′)-Ib* alone (42 isolates; 14.0%) and *aac(6′)-Ib* with *aac(3′)-IIa* (32 isolates; 10.7%) (Table 4). In addition, 23 of the isolates that harboured one or more AME gene also harboured an RMT gene.

Associations of AMEs, and AME combinations, with aminoglycoside MICs are shown in Table 5. Additionally all isolates non-susceptible to neomycin (*n* = 169) harboured the *aph(3′)-I* (*n* = 167) or/and the *aph(3′)-VI* (*n* = 5) gene. Among the 246 amikacin non-susceptible isolates, 214 (87.0%) encoded the *aac(6′)-Ib* gene, nine encoded the *aac(6′)-Ib* and the *aph(3′)-VI,* and 23 encoded the *rmtB* or the *armA* genes *(*±*aac(6′)-Ib)*. Among the 171 gentamicin non-susceptible isolates, 61 (35.7%) encoded the *aac(3′)-IIa* gene, two encoded the *ant(2′)-Ia* gene and 23 encoded the *rmtB (*±*aac(3′)-IIa)* or the *armA*. Twenty of the 254 isolates that harboured the *aac(6*′*)-Ib* gene and 12 of the 76 isolates that harboured the *aac(3′)-IIa* did not express phenotypic resistance to amikacin (MIC 2-8 mg/L) or gentamicin (MIC 1-2 mg/L), although those two genes confer resistance to amikacin and gentamicin, respectively.

PFGE genotyping revealed a multiclonal population of KPC-producing *K. pneumoniae*, with a prevalent PFGE profile (42.0%), consisting of nine variants, all detected in more than one center. Additionally, there were two more PFGE profiles identified, consisting of three and two variants each, and another 24 PFGE profiles that included a few isolates each.

The 21 VIM-, the 14 KPC- and VIM- and the 12 OXA-48-like - producing *K. pneumoniae* isolates were multiclonal, with no particular clone prevailing as different clones between hospitals and within hospitals were observed.

However, PFGE genotyping of NDM-producing isolates demonstrated great genetic similarity in the 48 (96.0%) isolates (dominant clone A), consisting of three main variants (A1 to A3), two of which were detected in more than one center. Additionally, two isolates, each with a distinct PFGE profile, were also detected (4.0%). Moreover, two isolates carrying both *bla*_NDM_ and *bla*_OXA-48-like_ belonged to dominant clone A.

The 23 *K. pneumoniae* isolates that harboured an RMT (*rmtB* or *armA*) and had highly elevated plazomicin MICs, belonged to six clonal types, suggesting that these isolates were not clonal, although clonal dissemination of *rmtB* positive VIM or KPC producing isolates was observed in three hospitals.

## Discussion

Among contemporary carbapenem-resistant *K. pneumoniae* isolates from Greece, where KPC-producing pathogens remain predominant, followed by NDM-producing isolates, plazomicin was more potent than that of the comparator aminoglycosides gentamicin and amikacin. These results are similar to those reported in previous studies with carbapenem-resistant enterobacteriaceae from diverse geographic regions [14–19].

There are several noteworthy findings in our study. We observed that in Greece, aminoglycoside resistance in carbapenemase-producing *K. pneumoniae* clinical isolates is predominantly caused by the production of AMEs (85.3% of isolates), while the occurrence of RMTs was observed in 7.7%. A remarkable AME diversity was observed. Overall, 23 different AME patterns (maximum of five genes/isolate) correlating with different levels of aminoglycoside resistance were identified.

The *aac(6′)-Ib* enzyme was the most common gene detected and found in ≥71.4% of carbapenemase producing strains, regardless of the carbapenemase present, whilst the *aac(3′)-IIa* gene was mainly associated with NDM- and OXA-48-producing isolates. The *ant(3′)-I* gene was always associated with VIM-, while *aph(3΄)-Ia* was mainly associated with KPC and VIM-producing *K. pneumoniae*.

Notably, the aminoglycoside resistance phenotype was not always a reliable predictor of the AME genotype. For instance, in 49.7% of the gentamicin non-susceptible isolates the *aac(3′)-IIa*, *ant(2′)-Ia, aac(3′)-Ia* or *aac(3)-IV* genes, were not detected, while *aac(3′)-IIa* was detected in 4% of the gentamicin susceptible isolates (MICs 1-2 mg/L). All amikacin non-susceptible isolates harbored the *aac(6′)-Ib* or an RMT gene, while 20 (6.7%) amikacin susceptible isolates harbored also the *aac(6′)-Ib* gene. This is consistent with previous studies where, despite the presence of *aac(6*′*)-Ib*, low amikacin MICs (2-8 mg/L) have been reported in *K. pneumoniae* and *E. coli* strains [16, 20, 21]. The contribution of multiple concurrent resistance mechanisms and differentiations in catalytic activity of AME genes is probably the explanation for this. There are 45 non-identical AAC(6′)-Ib-related entries in the NCBI database, with 1 to 8 amino acid differences and a total of 24 positions showing amino acid variations. Among them, 32 have identical name but a non-identical amino acid sequence (97-99.5% similarity). Some of these variants have conserved specificity, while others have not, i.e. AAC(6′)-Ib_11_ has an extended resistance spectrum that includes gentamicin or AAC(6′)-Ib’ confers resistance to gentamicin but not to amikacin [22]. On the contrary, the presence of high resistance (MIC ≥256 mg/L) to both amikacin and gentamicin correctly predicted (95.5%) the presence of an RMT gene, which also displayed similar highly elevated plazomicin MICs, which is consistent with the limitations of plazomicin and the aminoglycoside class.

There were 23 isolates that encoded both a carbapenemase and an RMT. Sixteen KPC- (8.0%), six VIM- (28.6%) and one KPC & OXA-48 -producing *K. pneumoniae* isolates harbored either *rmtB* or *armA*.

Acquired aminoglycoside resistance mediated by 16S-RMTases is a relatively new mechanism described in the early 2000s. Co-association of 16S-RMTases with carbapenemases leads to XDR and, in some instances, to PDR phenotypes [7].

In previous literature reports, plazomicin MICs were predominantly ≤4 mg/L, except for CRE isolates that produced the NDM-1 metallo-β-lactamase [15]. Interestingly, our findings showed that all 52 NDM-producing *K. pneumoniae* had plazomicin MICs ≤2 mg/L. This was similar to results found against NDM-producing Enterobacteriaceae from Brazil, which exhibited plazomicin MICs ≤4 mg/L [19]. In both countries, *bla*_NDM_ gene has been reported to be located on an IncFII-type plasmid [23–26], while aminoglycoside susceptibility was variable, suggesting that the mechanism of resistance was due to the presence of AMEs rather than 16S rRNA methyltransferase.

Plazomicin MICs in RMT-negative isolates were consistently lower than those of the other aminoglycosides, and further, the activity of plazomicin was not affected by the number or type of AMEs produced or by the presence of any carbapenemase. As plazomicin was designed to evade modifications conferred by most AMEs [14], these findings are not surprising.

Another noteworthy finding in our study was the apramycin susceptibility. Apramycin is a structurally unique aminoglycoside, a veterinary agent that has not been approved for clinical use, which is likely due to its narrow therapeutic index [15]. It is not inactivated by most of the known AMEs [27], and it is active against producers of the most common N7-G1405 RMTs [15]. Apramycin inhibited 86.7% of the *K. pneumoniae* isolates at ≤8 mg/L and it was the second most active drug after plazomicin. This is in accordance with previous evidence that apramycin has broad-spectrum activity against carbapenem-susceptible and carbapenem-resistant Enterobacteriaceae strains from the US, the UK and China [15, 28, 29], suggesting that apramycin may be a candidate for modification to potentially generate new potent aminoglycosides.

## Conclusions

In conclusion, plazomicin was active against most of the contemporary carbapenemase-producing *K. pneumoniae* isolates collected from 14 Greek hospitals, with 87.0% of the isolates inhibited by an MIC≤2 mg/L, while 94.2% of the isolates that did not carry a RMT gene were inhibited by an MIC≤2 mg/L. Plazomicin demonstrated the most potent in vitro inhibitory activity of all aminoglycosides (regardless of the AMEs produced) and of all other drugs typically used today to treat infections caused by such strains, suggesting that this agent may play an important role for the treatment of MDR *K. pneumoniae* infections. Dissemination of 16S-RMTases among already MDR organisms is an unwelcome event. Strict infection control measures have to be elaborated to prevent the spread of MDR organisms such as those described here that co-produced carbapenemases and RMTS.

## Additional file

Additional file 1: **Table S1.** Additional table includes sequences of primers used for simplex and multiplex PCRs for the detection of genes encoding aminoglycoside modifying enzyme and 16S rRNA methyltransferase genes in *Κ. pneumoniae.* (DOCX 19 kb)

## Abbreviations

AAC: N-acetyltransferase
AME: Aminoglycoside-modifying enzyme
CLSI: Clinical and Laboratory Standards Institute
ESBL: Extended-spectrum β-lactamase
EUCAST: European Committee on Antimicrobial Susceptibility Testing
EuSCAPE: European Survey on carbapenemase - producing Enterobacteriaceae
KPC: *Klebsiella pneumoniae* carbapenemase
MDR: Multi-drug resistant
MIC: Minimum inhibitor concentration
NARMS: National Antibiotic Resistance Monitoring Study
NCCLS: National Committee for Clinical Laboratory Standards
NDM: New Delhi metallo-β-lactamase
OXA: Oxacillinase
PCR: Polymerase chain reaction
PDR: Pan drug resistant
PFGE: Pulsed-field gel electrophoresis
QC: Quality control
R: Resistant
RMT: 16S rRNA methyltransferases
S: Susceptible
UPGMA: Unweighted pair-group method using arithmetic averages
VIM: Verona integron-encoded metallo-β-lactamase
XDR: Extensively drug resistant
