# Correction to: Nationwide epidemiology of carbapenem resistant *Klebsiella pneumoniae* isolates from Greek hospitals, with regards to plazomicin and aminoglycoside resistance

**DOI:** 10.1186/s12879-019-3854-1

**Published:** 2019-03-07

**Authors:** Irene Galani, Konstantina Nafplioti, Panagiota Adamou, Ilias Karaiskos, Helen Giamarellou, Maria Souli, Study Collaborators, Sofia Maraki, Viktoria Eirini Mavromanolaki, Vassiliki Papaioannou, Sofia Tsiplakou, Polyzo Kazila, Ioanna Diamanti, Nikoletta Charalampaki, Eleftheria Trikka-Graphakos, Marina Toutouza, Helen Vagiakou, Konstantinos Pappas, Anna Kyratsa, Angeliki Paschali, Konstantina Kontopoulou, Olga Legga, Efthymia Petinaki, Helen Papadogeorgaki, Efrosini Chinou

**Affiliations:** 1 Infectious Diseases Laboratory, 4th Department of Internal Medicine, National and Kapodistrian University of Athens, Faculty of Medicine, Athens, Greece; 2 6th Department of Internal Medicine, Hygeia Hospital, Athens, Greece; 3 University General Hospital “ATTIKON”, Rimini 1, 124 62 Chaidari, Greece

**Correction to: BMC Infectious Diseases 2019 19:167**

**https://doi.org/10.1186/s12879-019-3801-1**

Following publication of the original article [1], the authors reported that a collaborator’s name has been misspelled. Viktoria Eirini Mavromanolaki has been written by mistake Viktoria Eirini Mauromanolaki (u instead of v).
