# Supplementary material for: Nationwide epidemiology of carbapenem resistant Klebsiella pneumoniae isolates from Greek hospitals, with regards to plazomicin and aminoglycoside resistance
Source: BMC Infect Dis. 2019 Feb 15;19:167. doi: 10.1186/s12879-019-3801-1 (PMC6377745; doi:10.1186/s12879-019-3801-1)
Supplement: Supplementary file 1 — Table S1. Additional table includes sequences of primers used for simplex and multiplex PCRs for the detection of genes encoding aminoglycoside modifying enzyme and 16S rRNA methyltransferase genes in Κ. pneumoniae. (DOCX 19 kb) [file 12879_2019_3801_MOESM1_ESM.docx]

**Supplementary Table 1. Sequences of Primers Used for Simplex and Multiplex PCRs for Detection of Genes Encoding Aminoglycoside Modifying Enzyme and 16S rRNA Methyltransferase genes in** Κ. pneumoniae

| **PCR**  **name** | **Targeted gene** | **Primer name** | **Sequence (5' to 3' direction)** | **Amplicon size, bp** | **Reference** |
| --- | --- | --- | --- | --- | --- |
| **AAC(6’)-Ib** | aac(6’)-Ib | AAC(6’)-IF | TATGAGTGGCTAAATCGΑT | 395 | 1 |
|  |  | AAC(6’)-IR | CCCGCTTTCTCGTAGCA |  |  |
| **AAC(3’)-IIa** | aac(3’)-IIa | AAC(3’)-IIF | GCCGACTGGCACTGTGATGGGATAC | 359 | 2 |
|  |  | AAC(3’)-IIR | TGCAATGCGGTAACGGAGTTTAGCG |  |  |
| **AAC(3’)-Ia** | aac(3’)-Ia | AAC(3’)-IF | ACAAAGTTAGGTGGCTCAAGTATGGGCATC | 407 | 2 |
|  |  | AAC(3’)-IR | TCACCGTAATCTGCTTGCACGTAGATCAC |  |  |
| **AAC(3’)-IV** | aac(3’)-IV | AAC(3’)-IVF | CTCGAAGATGGGCCACTTGGACTGATC | 367 | 2 |
|  |  | AAC(3’)-IVR | AACTCGGCAAGATGCAGCGTCGTG |  |  |
| **APH(3')-VI** | *aph(3')-VI* | APH(3')-VIF | CGGAAACAGCGTTTTAGA | 716 | 3 |
|  |  | APH(3')-VIR | TTCCTTTTGTCAGGTC |  |  |
| **ANT(2’)-Ia** | ant(2’)-Ia | ANT(2’)-IF | ATGCGCTCACGCAACTGGTC | 749 | 4 |
|  |  | ANT(2’)-IR | GCATATCGCGACCTGAAAGC |  |  |
| **ANT(3’)-I** | *ant*(3’)-I | ANT(3’)-IF | GTGGATGGCGGCCTGAAGCC | 526 | 5 |
|  |  | ANT(3’)-IR | ATTGCCCAGTCGGCAGCG |  |  |
| **Multiplex 1** | *rmtA* | rmtAF | AAACTATTCCGCATGGTTC | 88 | 6 |
|  |  | rmtAR | TCATGTACACAAGCTCTTTCC |  |  |
|  | *rmtC* | rmtCF | CAGGGGTTCCAACAAGT | 246 |  |
|  |  | rmtCR | AGAGTATATAGCTTGAACATAAGTAGA |  |  |
|  | *rmtD* | rmtDF | TCGTTTCAGCACGTAAAACA | 652 |  |
|  |  | rmtDR | CAGCGCGAAATTCAAAAAGG |  |  |
|  | *rmtG* | rmtGF | ACGGAATGCCGCGCGAAGTA | 381 |  |
|  |  | rmtGR | TCTCCGCAAGCAGATCGCCG |  |  |
|  | *rmtH* | rmtHF | ATGACCATTGAACAGGCAGC | 464 |  |
|  |  | rmtHR | AGGGCAAAGGTAAAATCCCA |  |  |
| **Multiplex 2** | *armA* | armAF | ATTTTAGATTTTGGTTGTGGC | 101 | 6 |
|  |  | armAR | ATCTCAGCTCTATCAATATCG |  |  |
|  | *npmA* | npmAF | GGGCTATCTAATGTGGTG | 229 |  |
|  |  | npmAR | TTTTTATTTCCGCTTCTTCGT |  |  |
|  | *rmtB* | rmtBF | ACTTTTACAATCCCTCAATAC | 171 |  |
|  |  | rmtBR | AAGTATATAAGTTCTGTTCCG |  |  |
|  | *rmtE* | rmtEF | GATGCCGTGTCTGTTACGCCG | 446 |  |
|  |  | rmtER | ACGTGAACCCACGAGTCCTGC |  |  |
|  | *rmtF* | rmtFF | CGATCCTACTGGGCTCCAT | 314 |  |
|  |  | rmtFR | GGCATAGTGCTTTTCCATGC |  |  |

**References**

1. Ploy MC, Giamarellou H, Bourlioux P, Courvalin P, Lambert T. Detection of *aac(6')-I* genes in amikacin-resistant *Acinetobacter spp*. by PCR. Antimicrob Agents Chemother 1994; 38: 2925-8.
2. Aggen JB, Armstrong ES, Goldblum AA, Dozzo P, Linsell MS, Gliedt MJ, Hildebrandt DJ, Feeney LA, Kubo A, Matias RD, Lopez S, Gomez M, Wlasichuk KB, Diokno R, Miller GH, Moser HE. Synthesis and spectrum of the neoglycoside ACHN-490. Antimicrob Agents Chemother 2010; 54: 4636-42. doi: 10.1128/AAC.00572-10.
3. Noppe-Leclercq I, Wallet F, Haentjens S, Courcol R, Simonet M. PCR detection of aminoglycoside resistance genes: a rapid molecular typing method for *Acinetobacter baumannii*. Res Microbiol 1999; 150: 317-22
4. Dubois V, Arpin C, Dupart V, Scavelli A, Coulange L, André C, Fischer I, Grobost F, Brochet JP, Lagrange I, Dutilh B, Jullin J, Noury P, Larribet G,Quentin C. Beta-lactam and aminoglycoside resistance rates and mechanisms among *Pseudomonas aeruginosa* in French general practice (community and private healthcare centres). J Antimicrob Chemother 2008; 62: 316-23. doi: 10.1093/jac/dkn174.
5. Sadvang D, Aarestrup FM and Jensen LB. Characterization of integrons and antibiotic resistance genes in Danish multiresistant *Salmonella enterica* Typhimurium DT104. FEMS Microbiol Lett 1998; 160: 37–41.
6. Corrêa LL, Montezzi LF, Bonelli RR, Moreira BM, Picão RC. Revised and updated multiplex PCR, targeting acquired 16S rRNA methyltransferases. Int J Antimicrob Agents 2014; 43: 479-81. doi: 10.1016/j.ijantimicag.2014.02.003.
